# Premature ventricular contractions associated with isotretinoin use*

**DOI:** 10.1590/abd1806-4841.20165138

**Published:** 2016

**Authors:** Sevil Alan, Betül Ünal, Aytül Yildirim

**Affiliations:** 1 Akdeniz University School of Medicine – Antalya, Turkey

**Keywords:** Arrhythmias, cardiac, Isotretinoin, Tachycardia, ventricular

## Abstract

Isotretinoin has been considered a unique drug for acne treatment. However, it is associated with numerous adverse effects. Isotretinoin can trigger premature ventricular contractions. This report describes a 33-year-old-woman who presented with palpitations for 1 week while undergoing 1-month isotretinoin treatment for mild-moderate facial acne. An electrocardiogram and Holter monitoring showed premature ventricular contractions during isotretinoin (Roaccutane, Roche) treatment. Isotretinoin-related premature ventricular contractions were strongly suggested in this case due to the existence of documented premature ventricular contractions on electrocardiograms and the disappearance of these premature ventricular contractions two weeks after termination of the treatment To the authors' knowledge, there has been 1 reported case of premature ventricular contractions linked to isotretinoin use; this report describes a second such case.

## INTRODUCTION

Isotretinoin (13-cis-retinoic acid), a retinoic acid derivative, is the most effective drug in acne pathogenesis. It was first introduced in 1982 and has been used ever since to treat acne and it belongs to the first generation of synthetic 13-cis retinoic acid compounds.^1^ Isotretinoin is indicated for severe acne and moderate acne.^1^ The classic recommended dose is 0.5 to 1.0 mg/kg/day for 6 months. ^2^ The drug entails various side effects that impact on many systems in the body. The most common side effects are: chelitis, xerosis, ocular sicca, arthralgia, myalgia, headache and hyperlipidemia.^3^ Furthermore, there are reports on its adverse effects associated with the nervous, musculoskeletal, ocular, gastrointestinal, hematological, psychiatric, and cardiac systems.^3^ Most of the drug's side effects are probably predictable and dose-dependent, which has led to improvement in variable dose regimens.^2^ Unfortunately, rare but significant side effects (including depression, inflammatory bowel disease) may occur. Thus, careful monitoring is necessary to improve clinical outcomes and minimize potential adverse events.^4^ Systemic isotretinoin therapy may cause cardiac side effects on rare occasions, like atrial tachycardia, sinus tachycardia and congenital heart disease.^5^ The literature includes 1 reported case of PVCs linked to isotretinoin use.^6^ In this report, the authors describe a second case of premature ventricular contractions (PVCs) linked to isotretinoin use.

## CASE REPORT

**Figure 1:**
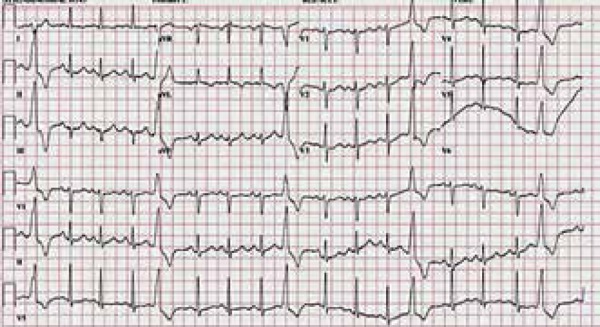
The patient's 12-lead ECG during isotretinoin use

A 35-year-old female presented with mild-moderate acne on her face. She started treatment with isotretinoin (Roaccutane^®^, Roche, Basel, Switzerland), at a dose of 30mg per day, for facial acne, which had lasted one month. No indications of smoking or alcohol use featured in the patient's personal history. Tests did not detect any other health problems, like hyperlipidemia, diabetes, hypertension and congenital, or acquired heart disease. Shortly after treatment was initiated, she complained of palpitations during exercise and rest. Before, she had never experienced any episodes of palpitations or other symptoms such as dizziness or chest pain. She consulted the Cardiology Department; her heart sounds were arrhythmic upon cardiovascular examination. A 12-lead electrocardiogram (ECG) showed premature ventricular contractions (PVCs) (Figure 1). A transthoracic echocardiography revealed a normal left and right ventricular size and function. Twenty-four-hour Holter monitoring showed frequent single, bigeminy, trigeminy, quadrigeminy uniform PVCs.

**Figure 2:**
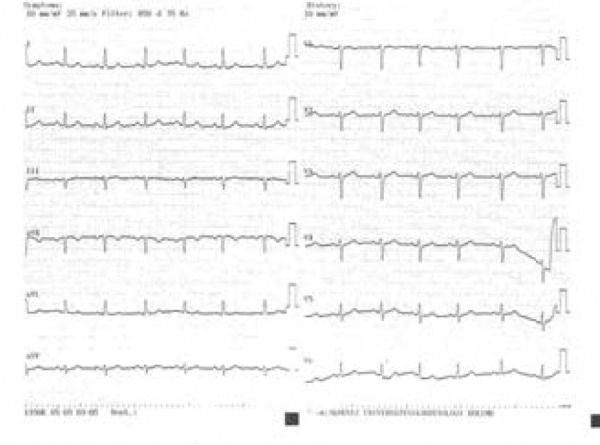
The patient's 12-lead ECG after discontinuation of the drug

When she was readmitted to the Cardiology Department two weeks after cessation of isotretinoin treatment, her cardiovascular examination was normal. There were no PVCs upon a 12-lead ECG and 24-hour Holter monitoring, or during exercise testing (Figure 2). She has remained asymptomatic since the discontinuation of the drug.

## DISCUSSION

Isotretinoin is used to treat moderate to severe acne cases that are unresponsive to conventional therapy.^4^ Several studies have been conducted to study the safety and efficacy of isotretinoin in treating moderate to severe acne vulgaris.^2,4,5^ According to case reports, systemic isotretinoin therapy can cause cardiac side effects, like atrial tachycardia, congenital heart disease, cardiac remodeling and sinus tachycardia.^5^ Charalabopoulos *et al.* described an 18-year old man who developed sinus tachycardia with transient right bundle branch block after three months of isotretinoin treatment.^7^ Güler E *et al* reported pericardial effusion with atrial tachycardia due to isotretinoin use.^5^. There are limited reports on the adverse cardiac effects of isotretinoin in literature. ^5,6^

Premature ventricular contractions (PVCs) are characterized by an early beat with a wide and abnormal QRS complex, without a preceding P wave.^6^ The T wave axis is usually across from the QRS. ^6^ PVCs can result from hypoxia, hypovolemia, electrolyte abnormalities, medications, or irritation from intracardiac monitoring or pacing catheters; it can also be idiopathic in nature.^6^ The therapeutic mechanism of retinoids and their side effects are not well defined.^7^ Large doses of retinol result in tissue damage due to the release of certain acid hydrolases and lysosomal enzymes into circulation.^8^ The excess of vitamin A causes considerable changes in heart electrogenesis (increased systole duration and decreased diastole duration), mainly because of alterations in myocardial cell membrane permeability.^9^ Cell membrane fragility for myocardial cells may provide a convincing explanation for the mechanism of PVCs.^9^ Structural changes in the cell membrane affect action potential, which may cause ventricular contractions.^9^ The authors believe that the temporal relationship between the isotretinoin treatment and the patient's documented arrhythmia on the ECG and Holter suggests a drug-induced cause.^6^ The effect of isotretinoin on ventricular rhythm cannot be discarded in this case. The report has highlighted a link between isotretinoin and documented PVCs. Hence, clinicians should be conscious of the possible arrhythmogenic effect(s) of isotretinoin and should be particularly careful with individuals suffering from underlying heart disease.
